# Trends in risk factors among young patients with acute myocardial infarction: a nationwide cohort study

**DOI:** 10.1093/ehjqcco/qcaf034

**Published:** 2025-05-26

**Authors:** Moa Simonsson, Moman A Mohammad, Sofia Sederholm Lawesson, Robin Hofmann, Jonas Faxén, David Erlinge, Christian Reitan, Pontus Andell

**Affiliations:** Department of Medicine, Solna, Karolinska Institutet, Stockholm, Sweden; Department of Cardiology, Karolinska University Hospital, Stockholm, Sweden; Department of Cardiology, Clinical Sciences, Lund University, Skåne University Hospital, Lund, Sweden; Department of Health, Medicine and Caring Sciences , Linköping University, Linköping, Sweden; Department of Cardiology, Linköping University, Linköping, Sweden; Department of Clinical Science and Education, Division of Cardiology, Karolinska Institutet, Södersjukhuset, Stockholm, Sweden; Department of Cardiology, Karolinska University Hospital, Stockholm, Sweden; Department of Physiology and Pharmacology, Karolinska Institutet, Stockholm, Sweden; Department of Cardiology, Clinical Sciences, Lund University, Skåne University Hospital, Lund, Sweden; Department of Clinical Sciences, Cardiology, Karolinska Institutet, Danderyd Hospital, Stockholm, Sweden; Department of Cardiology, Karolinska University Hospital, Stockholm, Sweden; Department of Physiology and Pharmacology, Karolinska Institutet, Stockholm, Sweden

**Keywords:** Acute myocardial infarction, Risk factor, Temporal trends, Young

## Abstract

**Aims:**

Acute myocardial infarction (AMI) is associated with significant loss-of-life expectancy, particularly among younger females. Addressing risk factors is crucial for optimizing prevention strategies. The aim of this study was to describe the trends in risk factors and clinical outcomes in a nationwide cohort of young AMI patients stratified by sex.

**Methods and results:**

Using Swedish registries, we included AMI patients aged 18–59 and selected five matched non-AMI controls from the general population. Risk factors included hypertension, diabetes, chronic kidney disease, hyperlipidaemia, obesity, smoking and systemic inflammatory disease. Clinical outcomes were major adverse cardiovascular events (death, AMI, stroke, or heart failure) and bleeding events. From 2006 to 2021, 44 254 AMI cases (9602 females and 34 652 males) and 220 721 non-AMI controls (47 967 females and 172 754 males) were included. Among AMI cases, the prevalence of at least one risk factor was higher in females than males (78.9 vs. 73.7%). Obesity increased from 25.2 to 35.5% becoming the most common risk factor. The risk of clinical outcomes was higher in cases than controls and in females compared with males among the cases, but lower among females compared with males among the controls. Five-year mortality was 6.1 vs. 1.3% in females and 4.9 vs. 1.8% males (cases vs. controls).

**Conclusion:**

Three in four young AMI patients have at least one known risk factor, of which obesity is the most common. Risk factor burden and clinical outcomes are worse among females. Prevention strategies and enhanced follow-up for young AMI patients should be prioritized. Further studies are needed to investigate sex-specific risk factors.

## Graphical Abstract

**Graphical Abstract:**
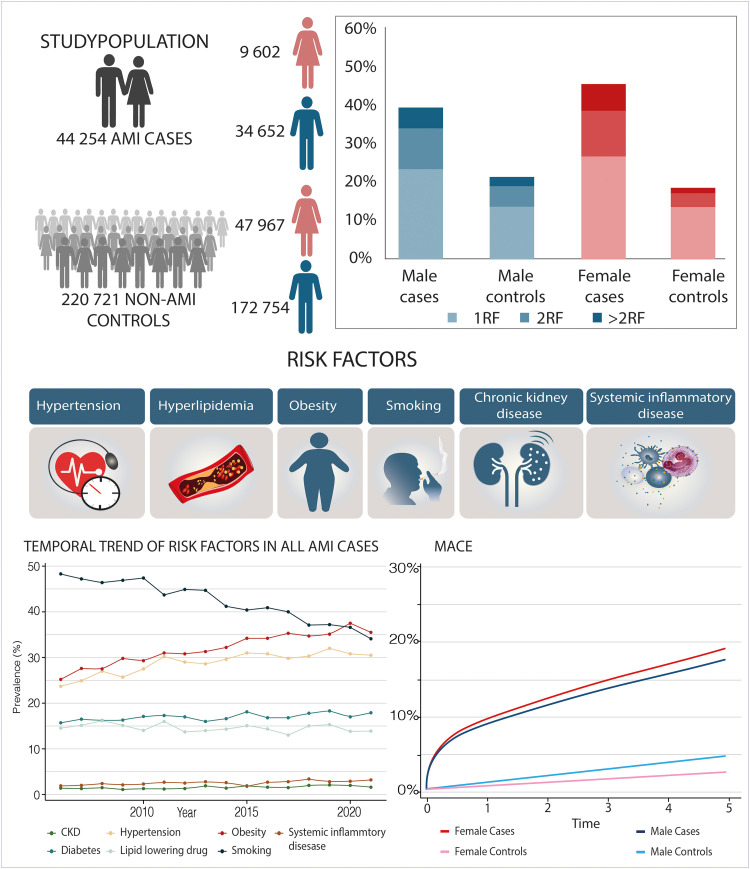

Key Learning Points What is already known:

- Addressing risk factors is crucial for improving both primary and secondary prevention.
- In younger individuals risk factor modification is particularly important due to longer exposure time and fewer competing prognostic risks.
- With growing global concern about unhealthy lifestyles, profiling the prevalence and trends of risk factors is of utmost importance.

What this study adds:

- In this study of a young AMI population, representive of an entire nation, from 2006 through 2021, we found a high prevalence of risk factors, particularly among females, and observed a rising prevalence of obesity.
- Compared with matched controls from the general population risk factors were significantly more common in individuals with AMI, with a particularly pronounced difference among females.
- Our findings underscore the urgent need for improved prevention strategies in younger individuals with AMI, with particular focus on young women, who exhibit higher risk burden. Further research is needed to better understand sex-specific risk factors and optimize prevention efforts.

## Introduction

Cardiovascular disease (CVD), including acute myocardial infarction (AMI) is the leading cause of death globally.^1^ The median age of patients hospitalized with a first-time AMI in Sweden is approximately 70 years, with females presenting about five years older than males.^2^ While the risk of AMI increases with age, its long-term consequences can be more severe in younger individuals,^3^ leading to greater loss of life expectancy,^4^ significant healthcare costs,^5^ and premature retirements, thereby imposing burdens on society. The loss of life expectancy has been shown to be greatest in young females with AMI.^4^

Previous studies have described the young AMI populations,^6^ including prevalence of the so-called traditional risk factors, such as hypertension, diabetes, smoking hyperlipidaemia and obesity,^3,7,8^ as well as sex-specific risk factors in different populations.^9,10^ However, many of these studies lack control populations^3,6-8,10^ or represent selected subpopulations.^9,10^ A comprehensive description of risk factors, stratified by sex, in young AMI patients is crucial for understanding the sex-related differences in outcomes and optimizing primary and secondary prevention strategies.

Our aim was to describe the temporal trends in AMI incidence, as well as the prevalence of risk factors and outcomes, in a nationwide cohort of young AMI patients, stratified by sex, compared with non-AMI controls from the general population.

## Methods

### Data sources

We used data from Swedish Web-system for Enhancement and Development of Evidence-based care in Heart disease Evaluated According to Recommended Therapies (SWEDEHEART),^11^ a national quality register including patients admitted with suspected AMI to a coronary care unit or other specialized inpatient facilities in Sweden. Overall, SWEDEHEART registers more than 90% of all patients treated with AMI in Sweden, with lowest coverage among older AMI patients who are treated on geriatric wards or outside the hospital setting. Thus, for younger individuals the coverage is likely close to 100%. We linked SWEDEHEART with the National Patient Register,^12^ the Swedish Population Register,^13^ the National Prescribed Drug Register^14^ and the Swedish longitudinal integrated database for health insurance and labour market studies (LISA).^15^ Details regarding data sources can be found in Supplementary material online.

This study was approved by the Swedish Ethical Review Authority (DNR: 2019–01458 and DNR 2012/60-13/2). According to Swedish law, written informed consent is not required when conducting register-based studies on pseudonymized data. The reporting followed the Strengthening of Observational Studies in Epidemiology (STROBE) reporting guideline.

### Study population

Patients aged 18–59 years, enrolled in the SWEDEHEART registry with a first-time AMI (ICD-10 code I21), from 1 January 2006, to 31 December 2021, were included. For each AMI patient, up to five non-AMI individuals were randomly selected from the Swedish Population Register at Statistics Sweden, matched by year of birth, sex, and geographical region of residence. Each non-AMI control could only be selected once but could later be included as a case if they experienced an AMI and were registered in SWEDEHEART.

### Definition of risk factors

Risk factors included hypertension, diabetes mellitus, chronic kidney disease (CKD), treatment with lipid-lowering drug, obesity, smoking, and systemic inflammatory disease. Hypertension was defined as either a prior, i.e. before the AMI admission, diagnosis of hypertension recorded in the National Patient Register or by the collection of a prescription for angiotensin converting enzyme inhibitors or angiotensin receptor blockers, without a diagnosis of heart failure, or calcium channel blockers, in the National Prescribed Drug Register within 180 days prior to admission. Diabetes mellitus was defined as either a prior diagnosis of diabetes mellitus recorded in the National Patient Register or the collection of a prescription for any glucose-lowering drug in the National Prescribed Drug Register within 180 days prior to admission. Chronic kidney disease was defined as a prior diagnosis of CKD recorded in the National Patient Register. Treatment with lipid-lowering drug was defined by the collection of a prescription for any lipid-lowering drug in the National Prescribed Drug Register within 180 days prior to admission. Obesity, defined as a body mass index (BMI) ≥ 30 kg/m^2^, and smoking status were defined as recorded in the SWEDEHEART registry and were thus only available for the cases. Smoking status is categorized in the SWEDEHEART registry as active smoker, former smoker, or never-smoker. In this study, only active smokers were classified as smokers. Systemic inflammatory disease was defined as any prior diagnosis of arthritis, vasculitis, or antiphospholipid syndrome, recorded in the National Patient Register. Details on the risk factors and variable definitions are provided in Supplementary material online, *Table S1*.

### Definition of outcome events

Cases and controls were followed for outcome events from admission up to 365 days and 5 years, respectively. All-cause death was identified from the Swedish Population Register. Ischaemic events were defined as re-hospitalization with AMI or ischaemic stroke. Heart failure events were defined as hospitalization with heart failure. Major adverse cardiovascular events (MACE) were defined as a composite of all-cause death, AMI, ischaemic stroke, or heart failure hospitalization. Bleeding was defined as hospitalization with an ICD-10 code of bleeding. A detailed description of the outcome events, including ICD-10 codes, is provided in the eSupplement (see Supplementary material online, *Table S2, S Table S3A* and *B*).

### Statistical analysis

We calculated the annual incidence of AMI by using the number of cases in the study population divided by total population estimates for Sweden, stratified on year, age, and sex. The population estimates were retrieved from the Statistics Sweden open data portal. (https://www.scb.se/vara-tjanster/oppna-data/ last assessed April 2024). The prevalence of risk factors was described for the entire study period and, for the AMI cases, also as a yearly temporal trend. Logistic regression was used to assess the association of risk factors with AMI occurrence. To better understand the prevention potential of individual risk factors we also calculated the adjusted population attributable fraction (PAF) for hypertension, diabetes mellitus, CKD, treatment with lipid-lowering drug. and systemic inflammatory disease. (Further description in Supplementary material online). In the logistic regression analysis and PAF calculation, obesity and smoking were not included as these variables were only available for the case group and not for the control population.

We evaluated clinical outcomes, including all-cause death, MACE, and bleeding at 365 days and 5 years. These outcomes were expressed as incidence proportions and incidence rates per 100 person-years and Kaplan–Meier curves were generated. In the time-to-event analyses, cases and controls were censored at the end of the study period (365 days or 5 years after admission), or the end of the observation period (May 2022). In the analysis of AMI incidence, cases were divided into age-groups and by sex (18–44 years female and male, 45–59 years female and male). In the analyses of risk factor prevalence and clinical outcomes, cases and controls were stratified by sex (female and male). A supplementary subgroup analysis of outcomes stratified by sex and ST-elevation myocardial infarction (STEMI), non-ST-elevation myocardial infarction (NSTEMI) classification was performed in the AMI-cases.

There were missing data in the SWEDEHEART-derived variables related to obesity and smoking status, and a negligible proportion missing for STEMI. Additionally, there were missing data in the income variable derived from LISA. The proportion of missing data is presented in *Table 1*. There were no missing data for the variables included in the logistic regression model. Missing data on the descriptive variables were not imputed.

**Table 1:** Baseline characteristics in acute myocardial infarction cases and non-acute myocardial infarction controls stratified by sex

	AMI cases *n* = 44 254		Non-AMI controls *n* = 220 721	
	Male *n* = 34 652	Female *n* = 9 602	Male *n* = 172 754	Female *n* = 47 967
**Demographics**				
Age (IQR)	54.0 (49.0–57.0)	54.0 (49.0–57.0)	54.0 (49.0–57.0)	54.0 (49.0–57.0)
BMI^a^ (kg/m^2^)	27.8(25.4–30.9)	27.4 (24.0–31.6)	N/A	N/A
Missing	3357 (9.7)	1022 (10.6)		
Obesity^a^ *n* (%) missing, please see BMI	9916 (28.6)	2888 (30.1)	N/A	N/A
Overweight^a^ missing, please see BMI	24 730 (71.3)	5761 (60.0)	N/A	N/A
BMI* > 27 Missing, please see BMI	18 846 (54.4)	4969 (51.8)	N/A	N/A
Smoking status^a^ *n* (%)			N/A	N/A
Active	13 749 (39.7)	4500 (46.9)		
Former	8194 (23.7)	1931 | (20.1)		
Never	11 714 (33.8)	2894 (30.1)		
Missing	995 (2.9)	277 (2.9)		
Disposable income category				
0	9480 (27.4)	1407(14.6)	36 835 (21.3)	4460 (9.3)
1	9553 (27.6)	2741 (28.6)	43 394 (25.1)	11 262 (23.5)
2	15 450 (44.6)	5430 (56.6)	91 927 (53.2)	32 121 (67.0)
Missing	169 (0.5)	24 (0.3)	598 (0.4)	124 (0.3)
Born outside Sweden	9627 (27.8)	2055 (21.4)	31 335 (18.1)	9029 (18.8)
STEMI^b^ *n* (%)	15 836 (45.7)	3589 (37.4)	N/A	N/A
Missing	54 (0.1)	16 (0.2)		
**Medical history**				
Hypertension *n* (%)	9564 (27.6)	3169 (33.0)	28 007 (16.2)	6308 (13.2)
Diabetes mellitus *n* (%)	5677 (16.4)	1828 (19.0)	9495 (5.5)	1650 (3.4)
Treatment with lipid lowering drug *n* (%)	5029 (14.5)	1434 (14.9)	14 511 (8.4)	2676 (5.6)
Previous PCI *n* (%)	829 (2.4)	152 (1.6)	763 (0.4)	51 (0.1)
Prior CABG *n* (%)	319 (0.9)	73 (0.8)	409 (0.2)	32 (0.1)
Prior stroke *n* (%)	659 (1.9)	270 (2.8)	2054 (1.2)	415 (0.9)
Prior bleeding *n* (%)	730 (2.1)	289 (3.0)	2743 (1.6)	730 (1.5)
Prior heart failure *n* (%)	413 (1.2)	144 (1.5)	946 (0.5)	124 (0.3)
Prior cancer *n* (%)	316 (0.9)	164 (1.7)	1356 (0.8)	600 (1.3)
Prior LEAD *n* (%)	340 (1.0)	165 (1.7)	604 (0.3)	128 (0.3)
CKD *n* (%)	497 (1.4)	194 (2.0)	832 (0.5)	130 (0.3)
COPD	541 (1.6)	400 (4.2)	536 (0.3)	235 (0.5)
Systemic inflammatory disease *n* (%)	619 (1.8)	505 (5.3)	1665 (1.0)	1188 (2.5)
**Medical treatment before admission**				
Aspirin *n* (%)	2962 (8.5)	868 (9.0)	6265 (3.6)	1095 (2.3)
P2Y12i *n* (%)	471 (1.4)	131 (1.4)	649 (0.4)	108 (0.2)
Betablocker *n* (%)	4633 (13.4)	1922 (20.0)	12 628 (7.3)	3724 (7.8)
RAAS *n* (%) not including ARNI	7433 (21.5)	2271 (23.7)	23 057 (13.3)	4625 (9.6)
RAAS *n* (%) including ARNI	7504 (21.7)	2239 (24.4)	23 274 (13.5)	4800 (10.0)
Caliciumblocker *n* (%)	3898 (11.2)	1174 (12.2)	11 309 (6.5)	2215 (4.6)
Diuretics *n* (%)	1939 (5.6)	1038 (10.8)	5175 (3.0)	2096 (4.4)
**Invasive treatment in-hospital**				
Angio^b^(%)	33 729 (97.3)	9187 (95.7)	N/A	N/A
PCI^b^ (%)	29 368 (84.8)	6516 (67.9)	N/A	N/A
CABG^b^ (%)	1526 (4.4)	253 (2.6)	N/A	N/A
Missing	11 084 (32.0)	3249 (33.8)		

AMI, Acute myocardial infarction; ARNI, angiotensinreceptor och neprilysin-inhibitor; BMI, body mass index; CABG, coronary artery bypass grafting; CKD, chronic kidney disease; COPD, chronic obstructive pulmonary diease; LEAD, lower extremity artery disease; N/A, not available, PCI, percutaneous coronary intervention; RAAS, renin–angiotensin–aldosterone system; STEMI, ST-elevation myocardial infarction.

^a^Data from the SWEDEHEART registry and not available for non-AMI controls.

^b^Data from the SWEDEHEART registry and not available or relevant for non-AMI controls.

Statistical analyses were performed in STATA MP version 15 (StataCorp. 2017. *Stata Statistical Software: Release 15*. College Station, TX: StataCorp LLC). Some data handling and plotting were performed with R version 4.2.2 and ggplot2 version 3.5.0.

## Results

From 1 January 2006, to 31 December 2021, a total of 208 773 patients with first-time AMI were enrolled in the SWEDEHEART registry with known admission and discharge date. Among these, 164 519 patients aged > 59 years were excluded, leaving 44 254 (9602 female and 34 652 male) patients aged 18–59 years as cases in the study. Additionally, 220 721 (47 967 female and 172 754 male) non-AMI individuals, matched by age, sex and geographical region of residence, served as controls(STROBE FLOW CHART and *Graphical abstract*).

**Figure:**
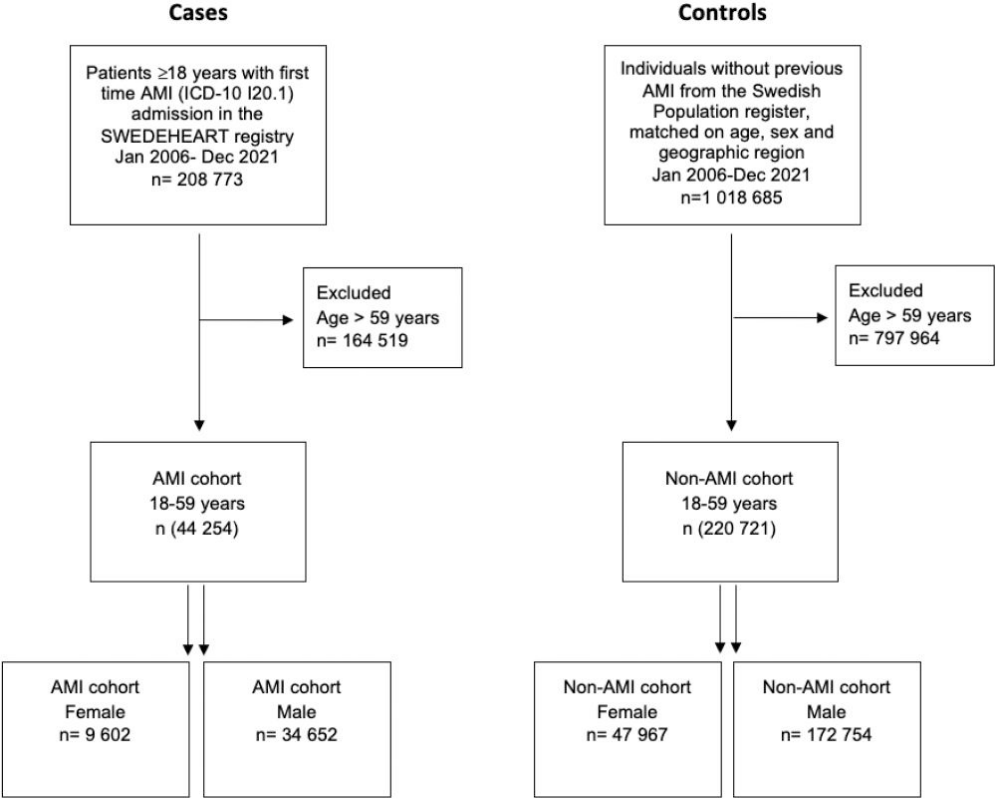


### Acute myocardial infarction incidence

**Figure 1:**
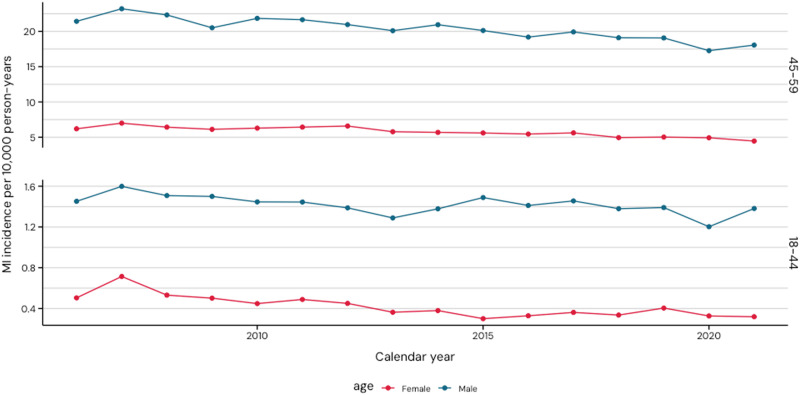
Incidence of acute myocardial infarction stratified by sex (female in red and male in blue) and age group (18–44 in the upper panel and 45–59 years in the lower panel).

The incidence of AMI was higher in patients aged 45–59 and was higher in males compared with females. Over the study period, the incidence of AMI declined across all groups in both males and females: from 21.4 to 18.0 per 10 000 person-years (py) among males aged 45–59 years, from 6.2 to 4.5 per 10 000 py among females aged 45–59 years, from 1.5 to 1.4 per 10 000 py among males aged 18–44 years and from 0.5 to 0.3 per 10 000 py among females aged 18–44 years. However, due to low incidence, the decrease in absolute numbers was small in the youngest age group (18–44 years). (*Figure 1* and see Supplementary material online, *Table S4*)

### Prevalence of risk factors among the acute myocardial infarction cases

The prevalence of risk factors was high among the AMI cases, with 33 108 (74.8%) having at least one risk factor, including hypertension, diabetes, treatment with lipid-lowering drug, CKD, obesity, smoking or systemic inflammatory disease. Female cases had a higher risk factor prevalence than males (any risk factor 78.9 vs. 73.7%). The most common risk factors were obesity, smoking and hypertension followed by diabetes and treatment with lipid-lowering drug. The prevalence of CKD and systemic inflammatory disease was low.

### Temporal trends of the prevalence of risk factors among acute myocardial infarction cases

**Figure 2:**
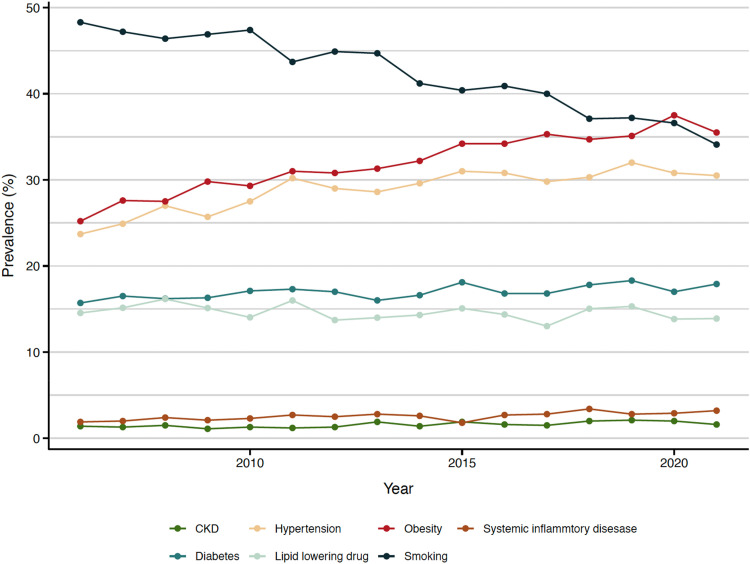
Prevalence of known prior risk factors from 2006 to 2021 among acute myocardial infarction patients 18–59 years.

While the prevalence of hypertension increased from an average of 25.2% in 2006–08 to 31.1% in 2019–2021, and diabetes increased from 16.1% in 2006–08 to 17.7% in 2019–21, the prevalence of treatment with lipid-lowering drug and CKD remained stable throughout the study period. The largest increase was observed in the prevalence of obesity, which increased from 25.2% in 2006 to 35.5% in 2021. At the start of the study period, smoking was the most common risk factor, with a prevalence of 48.2% in 2006, but it decreased to 34.1% by 2021. From 2020 onwards, obesity surpassed smoking to become the most common risk factor. (*Figure 2* and Graphical abstract) While the temporal trend was similar in female and male cases, female cases exhibited a higher prevalence of systemic inflammatory disease, hypertension, diabetes, obesity and smoking at nearly all time points. (see Supplementary material online, *Figure S2*).

### Prevalence of risk factors among acute myocardial infarction cases vs. controls

**Figure 3:**
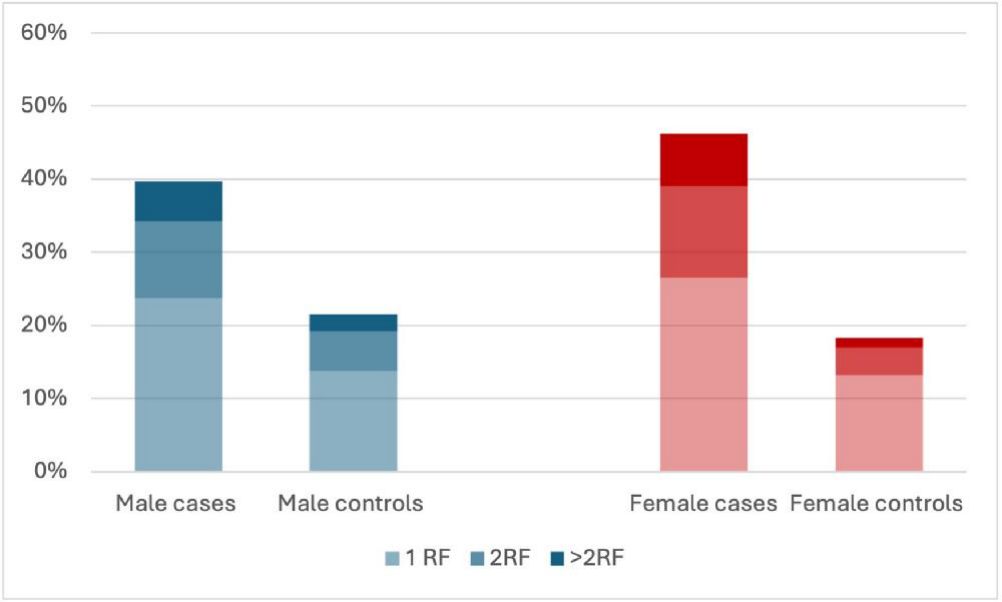
Prevalence of risk factors [hypertension, diabetes, hyperlipidaemia, chronic kidney disease and systemic inflammatory disease], divided into groups of one risk factor, 2 risk factors or more than two risk factors among acute myocardial infarction cases and non-acute myocardial infarction controls stratified by sex (men in blue and women in red). CKD, chronic kidney disease.

The prevalence of all risk factors (hypertension, diabetes, treatment with lipid-lowering drug CKD and systemic inflammatory disease) was higher in AMI cases than in controls (*Table 1* and *Table 2*, *Figure 3*), with a pronounced difference in female cases vs. female controls (hypertension 33.0 vs. 13.2%, diabetes mellitus 19.0 vs. 3.4%, treatment with lipid-lowering drug 14.9 vs. 5.6%, CKD 2.0 vs. 0.3% and systemic inflammatory disease 5.3 vs. 2.5%) (*Table 1*). When comparing males and females, the prevalence of risk factors was higher among female cases compared with male cases (*Figure 3*) (hypertension 33.0 vs. 27.6% diabetes mellitus 19.0 vs. 16.4%, treatment with lipid-lowering drug 14.9 vs.14.5%, CKD 2.0 vs. 1.4 and systemic inflammatory disease 5.3 vs. 1.8%). In contrast, the opposite was observed among the controls, with a lower prevalence of risk factors in female controls compared with male controls, (*Figure 3*) (hypertension 13.2. vs. 16.2% diabetes mellitus 3.4 vs. 5.5%, treatment with lipid-lowering drug 5.6 vs.8.4% and CKD 0.3 vs. 0.5%), except for systemic inflammatory disease which was more common in female controls (2.5 vs. 1.0%) (*Table 1*).

**Table 2:** Prevalence of risk factors among cases and controls

	AMI cases 44 254	Non-AMI controls^a^ *n* = 220 721
Hypertension *n* (%)	12 733 (28.8)	28 315 (12.8)
Diabetes Mellitus *n* (%)	7505 (17.0)	11 145 (5.0)
Treatment with lipid-lowering drug *n* (%)	6463 (14.6)	17 187 (7.8)
CKD n (%)	691 (1.6)	962 (0.4)
Systemic inflammatory disease *n* (%)	1124 (2.5)	2853 (1.3)
Obesity^a^ *n* (%)	12 804 (28.9)	N/A
Smoking status^a^ *n* (%)	18 249 (41.2)	N/A
Active	10 125 (22.9)	
Former	14 608 (33.0)	
Never	1272 (2.9)	

AMI, Acute myocardial infarction; CKD, chronic kidney disease; N/A, not available.

^a^ *Data on smoking and BMI was derived from the SWEDEHEART registry and not available for the non-AMI controls.*

### Population attributable fraction

The adjusted PAF was highest for diabetes (12.5%), and hypertension (11.5%) followed by systemic inflammatory disease (1.1%) and CKD (0.5%). Population attributable fraction was not calculated for treatment with lipid-lowering, as it was not significantly associated with AMI.

### Clinical outcomes at 365 days and 5 years

**Figure 4:**
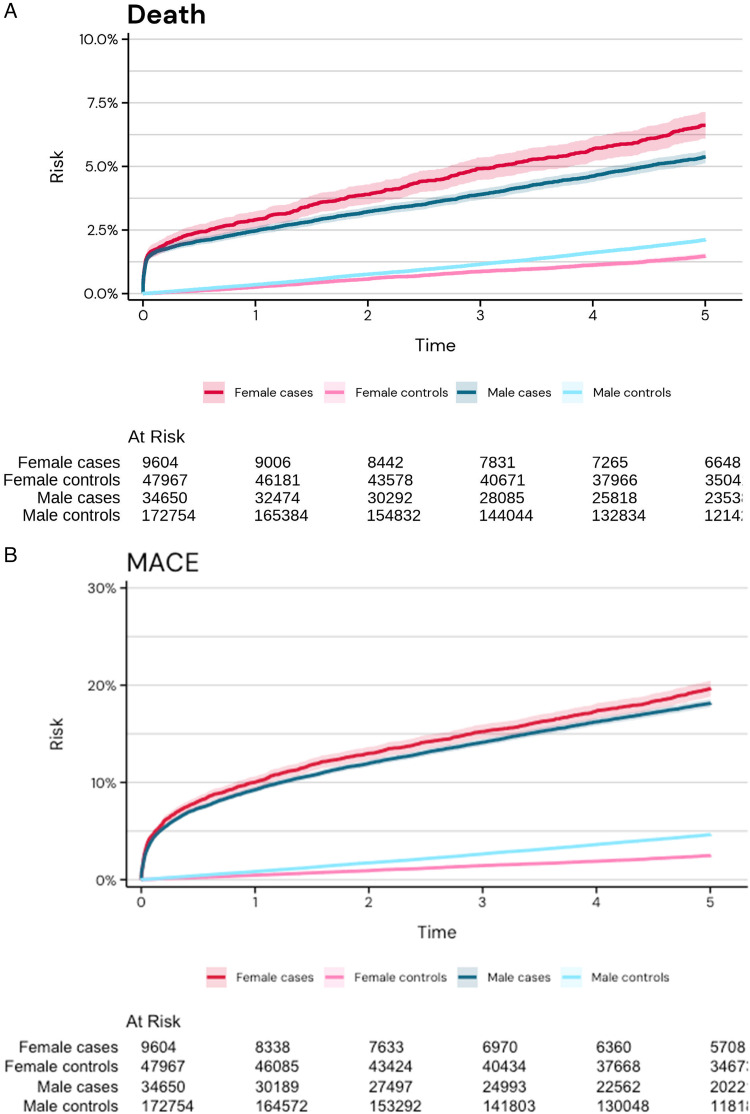
(*A*) Cumulative incidence of all-cause death in cases and controls stratified by sex (men in blue and females in red). (*B*) Cumulative incidence of major adverse cardiovascular events in cases and controls stratified by sex (men in blue and females in red). Major adverse cardiovascular events were defined as a composite of all-cause death, acute myocardial infarction, ischaemic stroke, or heart failure hospitalization. MACE, major adverse cardiovascular events.

The incidence of all-cause death at 365 days and 5 years was substantially higher in cases compared with controls: 2.5 vs. 0.3% and 5.2 vs. 1.7%, respectively. A similar pattern was observed for MACE and bleeding at both 365 days and 5 years (see Supplementary material online, *Table S5*, *Figure 4A* and *B*).

When comparing females and males, the incidence of all-cause death at 365 days and 5 years was numerically higher in female cases compared with male cases: 2.9 vs. 2.5% and 6.1 vs. 4.9%, respectively. A similar trend was observed for MACE and bleeding at 365 days and 5 years (*Table 3*, *Figure 4A* and *B*). However, among the controls, the incidence of clinical outcomes was numerically higher among males compared with females (*Table 3*).

**Table 3:** Outcomes at 365 days and 5 years in acute myocardial infarction cases and non-acute myocardial infarction controls stratified by sex

	AMI cases *N* = 44 254		Non-AMI controls *n* = 220 721	
	Male *n* = 34 652	Female *n* = 9 602	Male *n* = 172 754	Female *n* = 47 967
**Death 365 days**				
*n* (%)	852 (2.5)	278 (2.9)	597 (0.4)	122 (0.3)
Rate/100 py (CI 95%)	2.54 (2.38–2.72)	3.00 (2.66–3.37)	0.35 (0.32–0.38)	0.26 (0.22–0.31)
**Death 5 years**				
*n* (%)	1698 (4.9)	584 (6.1)	3139 (1.8)	618 (1.3)
Rate/100 person years (CI 95%)	1.18 (1.12–1.24)	1.45 (1.34–1.58)	0.43 (0.41–0.44)	0.29 (0.27–0.32)
**MACE 365 days**				
*n* (%)	3189 (9.2)	9578 (10.0)	1429 (0.8)	218 (0.5)
Rate/100 person years (CI 95%)	10.0 (9.67–10.37)	10.9 (10.24–11.62)	0.84 (0.80–0.89)	0.46 (0.40–0.52)
**MACE 5 years**				
*n* (%)	5800 (16.7)	1754 (18.3)	6910 (4,0)	1040 (2.2)
Rate/100 person years (CI 95%)	4.39 (4.28–4.51)	4.77 (4.55–5.00)	0.94 (0.92–0.96)	0.50 (0.47–0.53)
**Bleed 365 days**				
*n* (%)	672 (1.9)	202 (2.1)	455 (0.3)	127 (0.3)
Rate/100 person years (CI 95%)	2.03 (1.88–2.19)	2.20 (1.92–2.53)	0.27 (0.24–0.29)	0.27 (0.23–0.32)
**Bleed 5 years**				
*n* (%)	1304 (3.8)	427 (4.5)	2095 (1.2)	515 (1.1)
Rate/100 person years (CI 95%)	0.92 (0.87–0.97)	1.08 (0.98–1.19)	0.28 (0.27- 0.29)	0.25 (0.23- 0.27)

AMI, Acute myocardial infarction; MACE, major adverse cardiovascular events.

### Subgroup analysis of clinical outcomes in ST-elevation myocardial infarction and NSTEMI

The incidence of both all-cause death and MACE at 365 days and 5 years was significantly higher in patients with STEMI compared with those with NSTEMI, in both females and males. Additionally, event rates were numerically higher among females than males for both STEMI and NSTEMI, except for 5-year MACE, which was significantly higher in females than in males with NSTEMI. (see Supplementary material online, *Table S6* and *S* Supplementary material online, *Figure S3A* and *S3B*).

### Risk factors and associated risk of AMI

**Figure 5:**
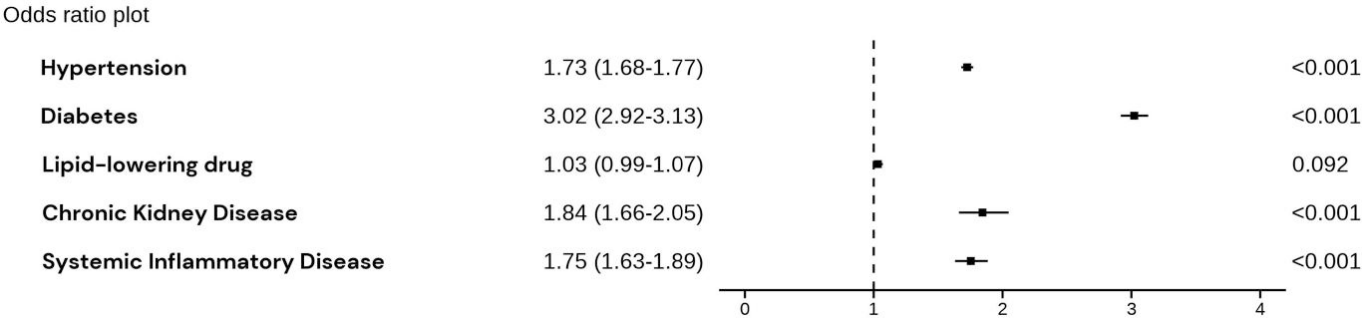
Risk factors and associated risk of myocardial infarction. Plot displaying odds ratios for a model adjusted for age and sex including the risk factors; hypertension, diabetes, treatment with lipid-lowering drugs, chronic kidney disease, and systemic inflammatory disease. Dashed line indicates OR 1 = no effect. CKD, chronic kidney disease.

In a multivariable logistic regression model that included traditional risk factors (hypertension, diabetes mellitus, treatment with lipid-lowering drug, CKD and systemic inflammatory disease), the strongest association with AMI was observed for diabetes mellitus [odds ratio (OR) 3.01 95% confidence interval (CI) 2.91–3.12] followed by CKD (OR 1.86 95% CI 1.68–2.07), systemic inflammatory disease OR 1.75, 95% CI 1.62–1.88) and hypertension OR 1.68, 95% CI 1.63–1.72). Treatment with lipid-lowering drug was not associated with MI OR (1.01 95% CI 0.98–1.05) (*Figure 5*).

## Discussion

In this nationwide study of young AMI patients and matched controls, the key findings were: (i) Risk factor prevalence remains high, with obesity increasing in prevalence and surpassing smoking to become the most common risk factor. (ii) Females have higher burden of risk factors and experience worse clinical outcomes than males. (iii) The greatest differences in risk factor burden and clinical outcomes were observed for female AMI cases and their matched controls, suggesting a particularly distinct phenotype among young females with AMI.

### The increasing burden of obesity and lifestyle-related risk factors

We found not only a high prevalence of risk factors, but also a rising prevalence of obesity, now affecting more than one-third of all AMI patients aged 18–59. This is concerning, though not surprising, as several reports have documented similar trends worldwide, highlighting obesity as a growing global public health concern with significant implications, both for individual health and for societal healthcare systems.^16^ Body mass index data were not available for the non-AMI controls, preventing direct comparison, but the Swedish Public Health Authority reports a similar, though less pronounced, trend of increasing overweight and obesity in the general population in Sweden (from 10.8 to 18.1% from 2004 until 2024 among individuals aged 16–84).^17^ Obesity is associated with cardiovascular disease including AMI, metabolic disorders such as Type 2 diabetes mellitus, hyperlipidaemia, and hypertension,^16^ several types of cancer and adverse effects on mental health. More recently, obesity has also been suggested to accelerate cardiovascular ageing.^18^

The most prevalent risk factors (obesity, smoking, hypertension, and diabetes) are either entirely lifestyle related (smoking), or at least partially influenced by lifestyle (obesity, hypertension, and diabetes) and potentially modifiable through life-style interventions and medications. In the recent European Society of Cardiology (ESC) Chronic Coronary Syndrome guidelines^19^ glucagone-like peptide-1 receptor agonists (GLP-1 RAs) received a Class IIa recommendation for patients with BMI ≥ 27 kg/m^2^. This implies that more than half of the young AMI population in our study may be eligible for GLP1-RA, irrespective of glycaemic status. However, implementation of this indication in Sweden is pending authorization from the European Medicines Agency and a reimbursement decision by national authorities. In addition to lifestyle interventions and medications, bariatric surgery^20^ is also an option for managing more pronounced obesity (BMI ≥ 35 kg/m^2^).

### Sex-related differences

Females generally have a lower incidence and later onset of CVD compared with males. In the Swedish population based SCAPIS study on approximately 30 000 females and males aged 50–65 years, males were found to develop coronary atherosclerosis 11–13 years earlier than females. Even after adjusting for both traditional and several non-traditional risk factors, including lifestyle and socioeconomic factors, males had a fourfold higher risk of coronary artery disease.^21,22^

Consistent with previous studies,^7,9^ we observed sex-related differences, with a higher burden of risk factors, and poorer prognosis in females compared with males.^23^This observation appears paradoxical given the generally longer life expectancy of females. It suggests that, for young to middle-aged females to experience atherosclerotic AMI, i.e. not primarily due to spontaneous coronary artery dissection (SCAD), a greater clustering of risk factors may be required compared with males of the same age.^24,25^

Moreover, it would be of great interest to investigate the presence of features associated with metabolic syndrome, such as central obesity and polycystic ovarian syndrome (PCOS), as well as gynaecological and obstetric history, including information on menopause, endometriosis, infertility, and pregnancy-related complications. These conditions^26,27,28^ have been associated with an increased risk of cardiovascular disease later in life, but such data were lacking in our study.

### Systemic inflammatory disease

Although the prevalence of systemic inflammatory disease was low, it may play a more significant role in driving vascular disease in younger individuals.^29^ Whether improved disease control or emerging immunomodulatory therapies can reduce the risk of vascular disease remains unclear. Furthermore, the role of primary prevention for cardiovascular events in this group of patients is not well established, as the definition of high-risk individuals typically includes only traditional risk factors, such as diabetes, hypertension, dyslipidaemia, and smoking.

### Population attributable fraction, modifiable risk factors and accumulated risk

The highest adjusted PAF was observed for diabetes (12.5%), followed by hypertension (11.5%), while the contributions of systemic inflammatory disease (1.1%) and CKD (0.5) were notably smaller, likely due to the lower prevalence of these conditions. These findings suggest that targeting modifiable risk factors, particularly diabetes and hypertension, could yield the greatest reduction of AMI incidence in this population.

Although younger individuals have a lower absolute risk compared with older individuals their accumulated risk over time is substantial.^30,31^ Optimizing prevention for these individuals is therefore crucial. This may include redistributing resources to enhance follow-up care of younger individuals after AMI, with intensified and prolonged surveillance in outpatient cardiology settings In addition, population-based primary preventive interventions to reduce the burden of risk factors is needed.

### Strengths and limitations

Despite the strengths of including the young AMI population of an entire nation, and comparison with matched controls, our study has several limitations. Data on BMI, smoking status, or laboratory data such as creatinine or cholesterol were not available in the National Patient Register. Cholesterol and familial hypercholesterolaemia (FH)^32,33^ are well-established risk factors for AMI and cardiovascular disease.^31,34^ Due to the lack of data on cholesterol and the introduction of the ICD-10 code for FH only after 2019, we used prescription of lipid-lowering drugs as a proxy. However, this is likely a poor indicator of actual cholesterol levels, which may explain the non-significant association between lipid-lowering treatment and AMI observed in our analysis. While Swedish national registers have demonstrated high validity,^12^ under-registration and misclassification of disease may still occur, but this would likely affect both cases and controls equally. Moreover, not all AMIs are caused by atherosclerotic disease. Conditions like myocardial infarction with non-obstructive coronary arteries, including SCAD^35^ may account for up to one third of AMIs in females under 50,^35^ but data on pathophysiology was lacking. Finally, we did not have data on illicit drug use, which has been identified as a relevant risk factor for AMI^7,36^

## Conclusion

The prevalence of risk factors among young AMI individuals is high, with approximately three in four having at least one known risk factor. Obesity has increased and is now the most common, followed by smoking and hypertension. Risk factor prevalence and clinical outcomes at 1 and 5 years are higher in females than males. These findings underscore the need for optimized primary prevention to reduce risk factor burden and AMI incidence, with particular focus on young women. Redistributing resources for secondary prevention post-AMI providing intensified and prolonged follow-up for younger individuals should be considered. Further observational studies are warranted to elucidate sex-related differences in risk factors, care and outcomes after AMI in younger individuals to guide improved prevention and follow-up.

## Supplementary Material

qcaf034_Supplementary_Data

## Data Availability

Due to data protection regulations the data will not be publicly available for external researchers.
