# Correction to: Trends in risk factors among young patients with acute myocardial infarction: a nationwide cohort study

**DOI:** 10.1093/ehjqcco/qcaf063

**Published:** 2025-07-21

This is a correction to: Moa Simonsson, Moman A Mohammad, Sofia Sederholm Lawesson, Robin Hofmann, Jonas Faxén, David Erlinge, Christian Reitan, Pontus Andell, Trends in risk factors among young patients with acute myocardial infarction: a nationwide cohort study, *European Heart Journal - Quality of Care and Clinical Outcomes*, Volume 11, Issue 6, September 2025, Pages 835–846, https://doi.org/10.1093/ehjqcco/qcaf034

The following changes have been made to the original publication. In section **Key learning points,** “from 2006 to 2022” has been emended to read: “from 2006 through 2021”.

