# Supplementary material for: Trends in risk factors among young patients with acute myocardial infarction: a nationwide cohort study
Source: Eur Heart J Qual Care Clin Outcomes. 2025 May 26;11(6):835–46. doi: 10.1093/ehjqcco/qcaf034 (PMC12445669; doi:10.1093/ehjqcco/qcaf034)
Supplement: qcaf034_Supplementary_Data [file qcaf034_Supplementary_Data.docx]

**Supplemental material**

**Young-MI**

**Description of data sources**

The SWEDEHEART (Swedish Web-system for Enhancement and Development of Evidence-based care in Heart disease Evaluated According to Recommended Therapies)^1^ registry collects information on demographics, admission logistics, baseline characteristics including past medical history and risk factors, electrocardiographic changes, biochemical markers medical treatment before admission, during hospital stay and at discharge, in-hospital interventions and outcomes and discharge diagnosis from all Swedish coronary care units (n=71). The diagnosis of myocardial infarction (MI) is determined by the physician responsible for treating the patient. The registry is monitored regularly, showing a 95-96% agreement between key variables in the registry and electronic health records.

The National Patient Register (NPR)^2^ includes all International Classification of Diseases (ICD) codes for all hospital admissions since 1987 and outpatient specialist care visits since 2001 but does not cover primary care visits. Since 1997 the NPR uses the tenth version, ICD-10. In our analysis, only hospital admissions were used to capture outcome events. The Swedish Population Registers^3^ hold information on major life events such as birth, death, marital status with close to full coverage for all births and deaths in Sweden. In our analyses, emigration could not be accounted for. The National Prescribed Drug Register holds information on all drug prescriptions filled at all pharmacies in Sweden since July 2005. The Swedish Longitudinal Integrated Database for Health Insurance and Labour Market Studies (LISA, Longitudinell Integrationsdatabas för Sjukförsäkrings- och Arbetsmarknadsstudier) covers the adult Swedish population aged ≥ 16 years registered on December 31 each year since 1990 (since 2010 individuals aged ≥ 15 years). The LISA database holds information on education, income, occupation, and employment by calendar year.

**S Table 1**

**Definition of baseline variables. SWEDEHEART definitions (only for cases) as defined at the index hospitalization for acute myocardial infarction. National patient registry for ICD-10 codes or corresponding ICD-9 codes (not shown).**

|  | **Data source, definitions and categorizations** |
| --- | --- |
| **Demographics** |  |
| Sex | SWEDEHEART:  Male or Female |
| BMI | As registered in SWEDEHEART calculated from weight and height  BMI was set as missing if extreme values on weight <30 kg or >199kg or extreme value on height <100, to account for incorrectly entered values in SWEDEHEART |
| Obesity | Derived from BMI as registered in SWEDEHEART  BMI $\geq$30  Extreme values on weight and height were truncated se above |
| Smoking status  Never  Former  Active | SWEDEHEART: Never  Former > 1 month Current smoker |
| Disposable income category*  0  1  2  Missing | The National Patient Register |
| STEMI | SWEDEHEART: No infarct N/A since only AMI cases included  NSTEMI  STEMI |
| **Medical history** |  |
| Hypertension | The National Patient Register:  ICD-10 code I10, I15  The National Prescribed Drug register:  Expedition of RAAS within 180 days prior to admission for AMI or entry as control, without a diagnosis of heart failure in the The National Patient Register  Expedition of calciumchannel blocker the The National Patient Register within 180 days prior to admission for AMI or entry as control |
| Diabetes Mellitus | The National Patient Register:  E10, E11, E12, E13, E14  The National Prescribed Drug register: Expedition of insulin or other glucose lowering drug* within 180 days prior to admission for AMI or entry as control |
| Treatment with lipid lowering drug | The National Prescribed Drug register: Expedition of lipid lowering drug within 180 days prior to admission for AMI or entry as control |
| Prior PCI | The National Patient Register:  FNG00 FNG01, FNG02, FNG03, FNG04, FNG05, FNG06 |
| Prior CABG | The National Patient Register:  FNA -FNF, FNH - FNW |
| Prior stroke | The National Patient Register:  I60, I61, I62, I63, I64 |
| Priorbleeding | The National Patient Register:  I60, I61, I62, 285B, 456A, 530H, 531A 531C 531E, 531G, 532A, 532C, 532E, 532G, 533A, 533C, 533E, 533G, 534A, 534C, 534E, 534G, 569D, D629, I850 K226, K250, K252, K254, K256, K260, K262, K264, K266, K270, K272, K274, K276, K280, K282, K284, K286, K290, K625, K920, K921, K922 |
| Prior heart failure | The National Patient Register:  I501, K761, I110 |
| Prior cancer | The National Patient Register:  C14 - C20 |
| Prior LEAD | The National Patient Register:  I70, I70, I72, I73 |
| CKD | The National Patient Register:  N17, N18, N19, N990, O084, I120, I13, I132, T795, P960 |
| COPD | The National Patient Register:  J40, J41, J42, J43, J44, J45, J46, J47 |
| Systemic inflammatory disease | The National Patient Register:  M05, M06, M07, M08, M09, M30, M32, M33, M34, M35, M36, M31, M310, M311, M313, M314, M315, M316, M317, M31, M31, M303, D686, D688 |
| **Medical treatment before admission** |  |
| Aspirin | The National Prescribed Drug register: Expedition of aspirin within 180 days prior to admission for MI or entry as control |
| P2Y12i | The National Prescribed Drug register: Expedition of P2Y12i within 180 days prior to admission for MI or entry as control |
| Betablocker | he National Prescribed Drug register:  Expedition of betablocker within 180 days prior to admission for MI or entry as control |
| RAAS | The National Prescribed Drug register  Expedition of ACEi or ARBi within 180 days prior to admission for MI or entry as control |
| Caliciumblocker | The National Prescribed Drug register  Expedition of calciumchannelblocker within 180 days prior to admission for MI or entry as control |
| Diuretics | The National Prescribed Drug register Expedition of diuretics within 180 days prior to admission for MI or entry as control |

ACE: angiotensin converting enzyme, AMI: Acute myocardial infarction, ARB: angiotensin receptor blockers  ARNI angiotensin receptor and neprilysin-inhibitor, BMI: Body Mass Index, CABG: coronary artery bypass grafting, CKD: chronic kidney disease, COPD: chronic obstructive pulmonary disease, LEAD: lower extremity artery disease, NSTEMI: Non-ST-elevation myocardial infarction PCI: percutaneous coronary intervention, RAAS: renin–angiotensin–aldosterone system, STEMI: ST-elevation myocardial infarction

*SGLT-2 inhibitors were approved for the treatment of heart failure in the European Union in November 2020, but did not receive reimbursement approval in Sweden until april 2021. Since only a negligible number (13 out of 15 777) of study participants were prescribed any per oral diabetes drug and had a diagnosis of heart failure in the National Patient Register 2021, we did not account for this potential source of misclassification.

**S Table 2 Definition of ischemic and heart failure events at one and five years**

| **One year event** | **Event (occurring within 365 days from discharge after the index AMI)** |
| --- | --- |
| AMI | Readmission for AMI registered in the SWEDEHEART registry (day 2-30 after discharge)  Readmission registered (day 31-365 after discharge) in the NPR with ICD10: I21 as primary diagnosis. |
| Ischemic stroke | Readmission (day 1-365 after discharge) registered in the National Patient Register with ICD10 I63 as primary or secondary diagnosis. |
| Heart failure | Readmission (day 1-365 after discharge) registered in the National Patient Register with ICD10 I63 as primary or secondary diagnosis. |
|  |  |
| **Five-year event** | **Event (occurring within 1825 days from discharge after the index AMI)** |
| AMI | Readmission for AMI registered in the SWEDEHEART registry (day 2-30 after discharge)  Readmission registered (day 31-1825 after discharge) in the NPR with ICD10: I21 as primary diagnosis. |
| Ischemic stroke | Readmission (day 1-1825 after discharge) registered in the National Patient Register with ICD10 I50, K761, I971, I1130, 1132 as primary or secondary diagnosis. |
| Heart failure | Readmission (day 1-1825 after discharge) registered in the National Patient Register with ICD10 I50, K761, I971, I1130, I1132 as primary or secondary diagnosis. |

AMI: acute myocardial infarction, ICD 10: International Statistical Classification of Diseases and Related Health Problems - Tenth Revision, NPR: National Patient Register, SWEDEHEART: Swedish Web-system for Enhancement and Development of Evidence-based care in Heart disease Evaluated According to Recommended Therapies

We did not include AMI events registered in SWEDEHEART during the first 2 days after discharge from the index AMI and AMI events registered in NPR within 30 days after discharge after the index AMI. These event definitions were chosen to lower the risk of counting events related to the index AMI that do not constitute a new event. Specifically, in NPR, diagnoses of AMI may be registered on several occasions during the patient’s hospitalization for their index AMI. This may occur when a patient is transferred from the cardiology clinic to a rehabilitation clinic or when the patient is rehospitalized shortly after discharge even if the rehospitalization is not due to a new AMI event.

In SWEDEHEART, the risk of such multiple entries of the same AMI is much lower. However, it may happen that the same AMI is registered twice if the patient is moved between cardiology clinics during the acute phase of the AMI. Therefore, we did not consider new AMI diagnoses registered in SWEDEHEART during the first 2 days after discharge from the index AMI.

**S Table 3a Definition of bleeding events**

| Bleeding at one year | Readmission (day 1-3655 after discharge) registered in the National Patient Register with ICD10 code of bleeding, as listed below, as primary or secondary diagnosis. |
| --- | --- |
| Bleeding at 5 years | Readmission (day 1-1825 after discharge) registered in the National Patient Register with ICD10 code of bleeding, as listed below, as primary or secondary diagnosis. |

**S Table 3b ICD-10 codes of bleeding**

| **ICD-10 code** | **Diagnosis** | **Type of bleeding** |
| --- | --- | --- |
| I60 | Subarachnoidal bleeding | Intracranial |
| I61 | Intracerebral bleeding | Intracranial |
| I62 | Other intracranial bleeding | Intracranial |
| D629 | Anemi after acute larger bleeding | Other bleeding |
| D500 | Iron deficiency anemia secondary to chronic blood loss | Other bleeding |
| H356 | Retinal bleeding | Other bleeding |
| H431 | Vitreous bleeding | Other bleeding |
| H450 | Vitreous bleeding | Other bleeding |
| H922 | Bleeding from the ear | Other bleeding |
| I850 | Esophageal varices with bleeding | GI-bleeding |
| K226 | Gastro-esophageal ulcer with bleeding, Mallory Weiss | GI-bleeding |
| K250 | Ulcus ventriculi with bleeding | GI-bleeding |
| K252 | Ulcus ventriculi with bleeding | GI-bleeding |
| K254 | Ulcus ventriculi with bleeding | GI-bleeding |
| K256 | Ulcus ventriculi with bleeding | GI-bleeding |
| K260 | Ulcus duodeni with bleeding | GI-bleeding |
| K262 | Ulcus duodeni with bleeding | GI-bleeding |
| K264 | Ulcus duodeni with bleeding | GI-bleeding |
| K266 | Ulcus duodeni with bleeding | GI-bleeding |
| K270 | Ulcus ventriculi or duodeni with bleeding | GI-bleeding |
| K272 | Ulcus ventriculi or duodeni with bleeding | GI-bleeding |
| K274 | Ulcus ventriculi or duodeni with bleeding | GI-bleeding |
| K276 | Ulcus ventriculi or duodeni with bleeding | GI-bleeding |
| K280 | Reccurence of bleeding ulcer after op | GI-bleeding |
| K282 | Reccurence of bleeding ulcer after op | GI-bleeding |
| K284 | Reccurence of bleeding ulcer after op | GI-bleeding |
| K286 | Reccurence of bleeding ulcer after op | GI-bleeding |
| K290 | Acute hemorrhagic gastritis | GI-bleeding |
| K625 | Bleeding in anus or rectum | GI-bleeding |
| K920 | GI-bleeding | GI-bleeding |
| K921 | GI-bleeding | GI-bleeding |
| K922 | GI-bleeding | GI-bleeding |
| N421 | Prostate bleeding | Urogenital bleeding |
| N938 | Bleeding from uterus or vagina | Urogenital bleeding |
| N939 | Bleeding from uterus or vagina | Urogenital bleeding |
| N950 | Bleeding after menopause | Urogenital bleeding |
| R041 | Bleeding from pharynx | Urogenital bleeding |
| R048 | Bleeding from the airways | Urogenital bleeding |
| R049 | Bleeding from the airways | Urogenital bleeding |
| R319 | Hematuria | Urogenital bleeding |
| T810 | Bleeding complicating a procedure | Other bleeding |
| N501A | Bleeding from male genitalia | Urogenital bleeding |

**S Table 4 Incidence per 10 000 person-years of MI stratified by age group and sex**

|  | Age 18-44 | | Age 45-59 | |
| --- | --- | --- | --- | --- |
| Year | Female | Male | Female | Male |
| 2006 | 0.50 | 1.45 | 6.19 | 21.43 |
| 2007 | 0.71 | 1.60 | 6.99 | 23.21 |
| 2008 | 0.53 | 1.51 | 6.42 | 22.33 |
| 2009 | 0.50 | 1.50 | 6.12 | 20.51 |
| 2010 | 0.45 | 1.45 | 6.28 | 21.85 |
| 2011 | 0.49 | 1.44 | 6.43 | 21.66 |
| 2012 | 0.45 | 1.39 | 6.58 | 20.96 |
| 2013 | 0.36 | 1.29 | 5.78 | 20.10 |
| 2014 | 0.38 | 1.38 | 5.68 | 20.95 |
| 2015 | 0.30 | 1.49 | 5.60 | 20.12 |
| 2016 | 0.33 | 1.41 | 5.45 | 19.19 |
| 2017 | 0.36 | 1.46 | 5.61 | 19.92 |
| 2018 | 0.34 | 1.38 | 4.94 | 19.10 |
| 2019 | 0.40 | 1.39 | 5.02 | 19.07 |
| 2020 | 0.33 | 1.20 | 4.92 | 17.27 |
| 2021 | 0.32 | 1.38 | 4.45 | 18.06 |

MI: myocardial infarction

**S Table 5. Outcomes at 365 days and 5 years in AMI cases and non-AMI controls**

|  | **AMI cases**  **44 254** | **Non-AMI controls**  **n=220 721** |
| --- | --- | --- |
| **Death 365d**  n (%)  Rate/100 py (CI 95%) | 1 130 (2.5%) | 719 (0.3) |
| **Death 5y**  n (%)  Rate/ 100 person years (CI 95%) | 2 282 (5.2) | 3 757(1.7) |
| **MACE 365d**  n (%)  Rate/ 100 person years (CI 95%) | 4 147 (9.4) | 1 647(0.07) |
| **MACE 5y**  n (%)  Rate/ 100 person years (CI 95%) | 7 734 (17.5) | 7 950 (3,6%) |
| **Bleed 365d**  n (%)  Rate/ 100 person years (CI 95%) | 874 (2.0) | 582 (0.3) |
| **Bleed 5y**  n (%)  Rate/ 100 person years (CI 95%) | 1 713(3.9) | 2 610 (1.1) |

AMI: Acute myocardial infarction, MACE: major adverse cardiovascular events

**S Table 6 Outcomes at 365 days and 5 years stratified by sex and STEMI/NSTEMI**

|  | **STEMI***  **N=19 425** | | **NSTEMI***  **n=24 759** | |
| --- | --- | --- | --- | --- |
|  | **male**  **n= 15 836** | **female**  **n= 3 589** | **male**  **n=18 762** | **female**  **n=5997** |
| **Death 365d**  n (%)    Rate/100 py (CI 95%) | 510 (3.2)  3.40 (3.12 - 3.71) | 142 (4.09)  4.19 (3.55 - 4.94) | 340 (1.8)  1.88 (1.69 - 2.09) | 134 (2.2)  2.32 (1.96 - 2.75) |
| **Death 5y**  n (%)    Rate/ 100 person years (CI 95%) | 853 (5.4)  1.31 (1.23 - 1.40) | 256 (7.1)  1.72 (1.52 - 1.95) | 840 (4.5)  1.08 (1.01 - 1.15) | 323 (5.4)  1.29 (1.16 - 1.44) |
| **MACE 365d**  n (%)    Rate/ 100 person years (CI 95%) | 1 759 (11.1)  12.33 (11.76 - 12.91) | 436 (12.2)  13.59 (12.37 - 14.93) | 1 417 (7.6)  8.08 (7.67 - 8.51) | 515 (8.6)  9.26 (8.50 - 10.10) |
| **MACE 5y**  n (%)    Rate/ 100 person years (CI 95%) | 2 942 (18.6)  4.99 (4.81 - 5.17) | 717 (20.0)  5.30 (4.93 - 5.71) | 2 819 (15.0)  3.87 (3.73 - 4.02) | 1 021 (17.0)  4.41 (4.14 - 4.68) |
| **Bleed 365d**  n (%)    Rate/ 100 person years (CI 95%) | 298 (1.9)  1.99 (1.77 - 2.22) | 62 (1.7)  1.83 (1.42 - 2.34) | 374 (2,0)  2.07 (1.87 - 2.29) | 139 (2.3)  2.42 (2.05 - 2.85) |
| **Bleed 5y**  n (%)    Rate/ 100 person years (CI 95%) | 550 (3.5)  0.85 (0.78 - 0.93) | 140 (3.9)  0.95 (0.80 - 1.12) | 750 (4.0)  0.97 (0.90 - 1.04) | 286 (4.8)  1.16 (1.03 - 1.30) |

AMI: Acute myocardial infarction, MACE: major adverse cardiovascular events, STEMI: ST-elevation myocardial infarction, NSTEMI: non-ST elevation myocardial infraction

*70 had missing on STEMI/NSTEMI status and were excluded in these analyses

**Calculation of Population attributable Fraction (PAF)**

Population attributable fraction (PAF) estimates the proportion of a disease or outcome in a population that could be attributed to a specific risk factor. It reflects the potential reduction in the outcome that would occur if the risk factor were eliminated, assuming all other factors remain constant. PAF incorporates both the prevalence of the risk factor and the strength of its association with the outcome. It is relevant and can help inform healthcare planning and allocation of resources.

Since the disease (AMI) is uncommon (< 10%) in the population, relative risk (RR) can be replaced by the Odds Ratio (OR) and calculated by the formula:


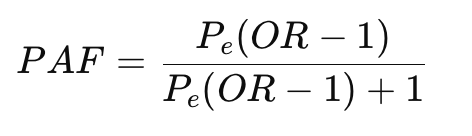


P_e_​= prevalence of the riskfactor
OR_adj=_ OR adjusted for age, sex, hypertension, diabetes, treatment with lipidlowering, CKD and autoimmune disease

Adjusted PAF was not calculated for treatment with lipid lowering as it was not significantly associated with AMI.

**Hypertension**P_e_= 17,8%. OR_adj_= 1.73

$\frac{0.12994}{1.12994}$=0,11499

**Diabetes**P_e_= 7.04%. OR_adj_= 3.02

$\frac{0.1422087}{1.1422087}$ =0.1245026412

**CKD**P_e_= 0.62%. OR_adj_= 1.84

$\frac{0.005208}{1.005208}$ =0.00518

**Autoimmune**P_e_= 1.5%. OR_adj_= 1.75

$\frac{0.005208}{1.005208}$ =0,0111

**S Figure 1**

Number of AMI cases per year divided by age groups, 18-44 years, 45-59 years and > 59 years

**S Figure 2**


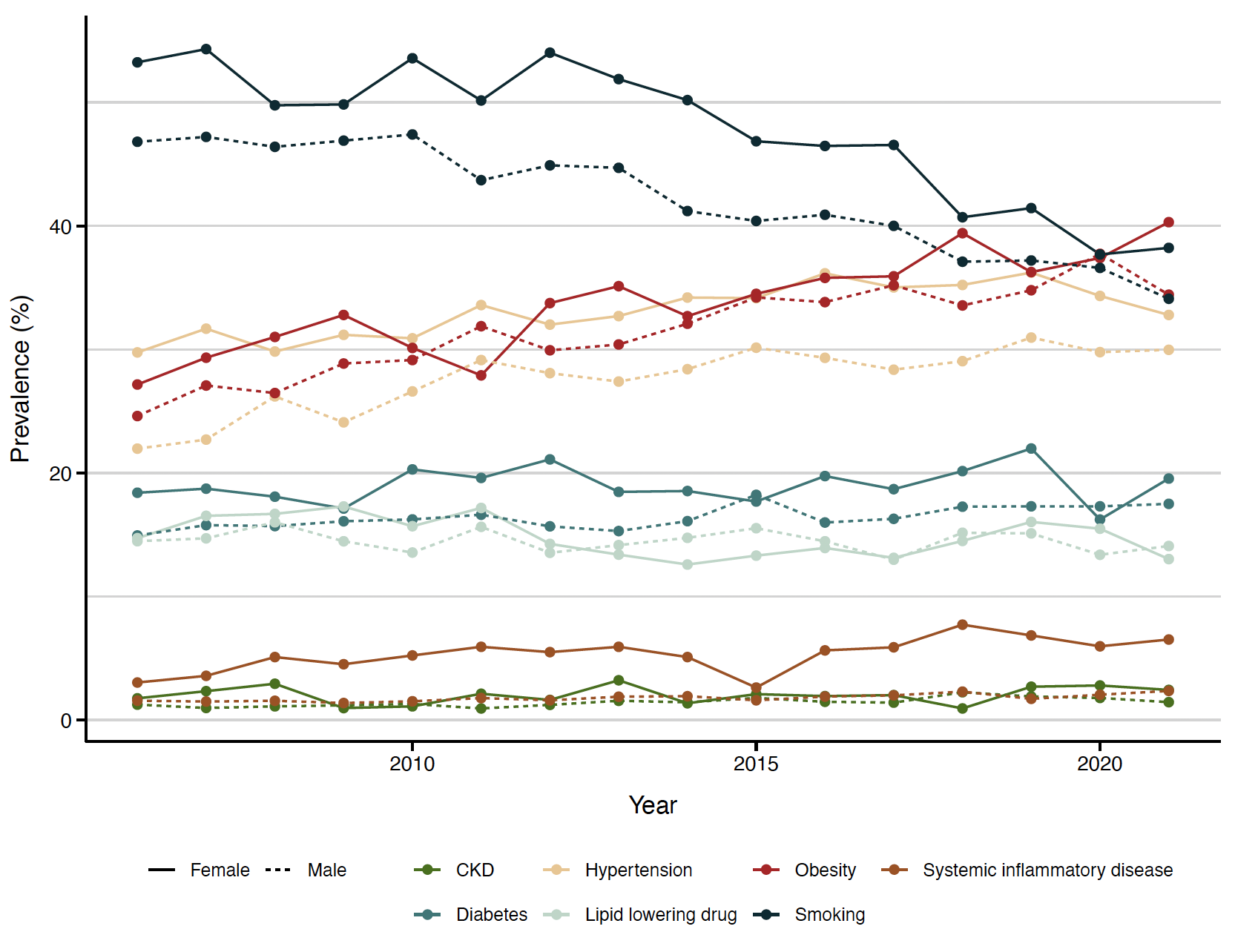


Prevalence of known prior risk factors from 2006 to 2021 among AMI patients 18-59 years stratified by sex. Females in solid lines and males in dotted lines.

**S Figure 3a**


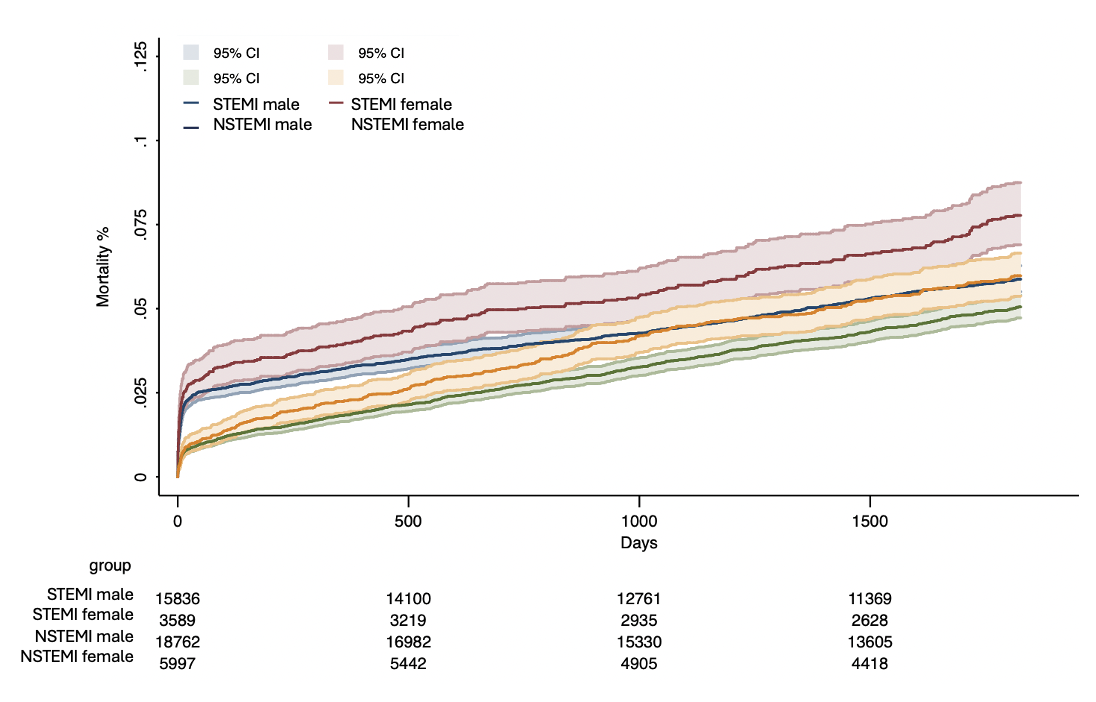


Cumulative incidence of mortality in female and male patients with STEMI and NSTEMI

**S Figure 3b**


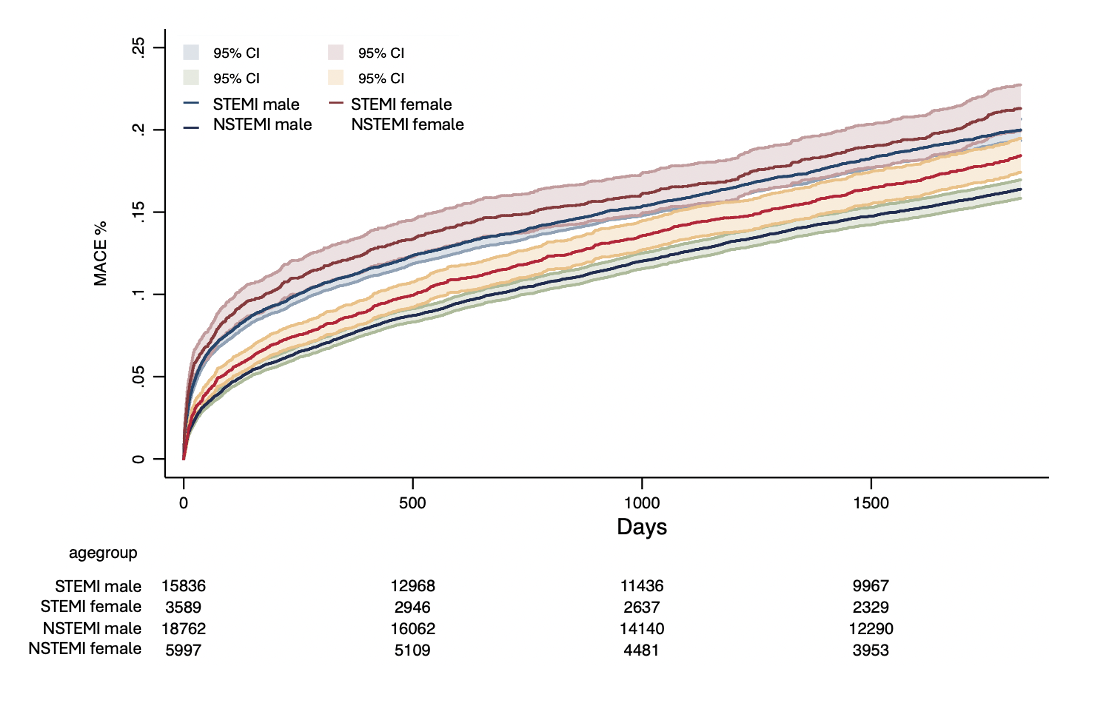


Cumulative incidence of a major adverse cardiovascular events (composite of all-cause death, AMI, ischemic stroke, or heart failure hospitalization) in female and male patients with STEMI and NSTEMI.
